# *catena*-Poly[[[bis­[4-(1*H*-1,3,7,8-tetra­azacyclo­penta­[*l*]phenanthren-2-yl)­phenol-κ^2^ *N* ^7^,*N* ^8^]lead(II)]-μ-4,4′-oxy­dibenzoato-κ^3^ *O*,*O*′:*O*′′] dihydrate]

**DOI:** 10.1107/S1600536808010805

**Published:** 2008-04-23

**Authors:** Mao-Liang Xu, Rui Zhou, Ge-Yang Wang, Seik Weng Ng

**Affiliations:** a Xi’an Modern Chemistry Research Institute, Xi’an 710065, People’s Republic of China; b Department of Chemistry, University of Malaya, 50603 Kuala Lumpur, Malaysia

## Abstract

The carboxyl­ate dianion in the title compound, [Pb(C_14_H_8_O_5_)(C_19_H_12_N_4_O)_2_]·2H_2_O, uses one carboxyl­ate group to *O*,*O*′-chelate a bis­[4-(1*H*-1,3,7,8-tetra­azacyclo­penta­[*l*]phen­anthren-2-yl)phenol]-chelated Pb^II^ atom and uses its other carboxyl­ate group to bind to another Pb^II^ atom in an irregular monodentate manner. The Pb^II^ atom exists in an undefined seven-coordinate geometry in the chain structure; the lone pair is stereochemically active. Adjacent chains are linked by inter­molecular O—H⋯N, N—H⋯O and O—H⋯O hydrogen bonds that involve the uncoordinated water mol­ecules to form a three-dimensional network.

## Related literature

For a transition metal dicarboxyl­ate adduct of 4-(1*H*-1,3,7,8-tetra­azacyclo­penta­[*l*]phenanthren-2-yl)phenol, see: Xu *et al.* (2008 ▶).
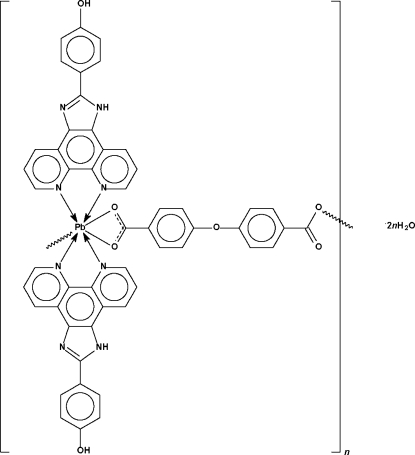


## Experimental

### 

#### Crystal data

[Pb(C_14_H_8_O_5_)(C_19_H_12_N_4_O)_2_]·2H_2_O
*M* *_r_* = 1124.08
Monoclinic, 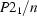

*a* = 10.767 (4) Å
*b* = 29.916 (7) Å
*c* = 13.688 (4) Å
β = 97.70 (1)°
*V* = 4369 (2) Å^3^
*Z* = 4
Mo *K*α radiation
μ = 3.93 mm^−1^
*T* = 295 (2) K
0.33 × 0.24 × 0.21 mm

#### Data collection

Rigaku R-AXIS RAPID diffractometer
Absorption correction: multi-scan (*ABSCOR*; Higashi, 1995 ▶) *T*_min_ = 0.188, *T*_max_ = 0.492 (expected range = 0.167–0.438)
42355 measured reflections
9977 independent reflections
6235 reflections with *I* > 2σ(*I*)
*R*_int_ = 0.063

#### Refinement

*R*[*F* ^2^ > 2σ(*F* ^2^)] = 0.054
*wR*(*F* ^2^) = 0.168
*S* = 1.02
9977 reflections
631 parameters
H-atom parameters constrained
Δρ_max_ = 1.51 e Å^−3^
Δρ_min_ = −1.09 e Å^−3^

### 

Data collection: *RAPID-AUTO* (Rigaku, 1998 ▶); cell refinement: *RAPID-AUTO*; data reduction: *CrystalStructure* (Rigaku/MSC, 2002 ▶); program(s) used to solve structure: *SHELXS97* (Sheldrick, 2008 ▶); program(s) used to refine structure: *SHELXL97* (Sheldrick, 2008 ▶); molecular graphics: *X-SEED* (Barbour, 2001 ▶); software used to prepare material for publication: *publCIF* (Westrip, 2008 ▶).

## Supplementary Material

Crystal structure: contains datablocks global, I. DOI: 10.1107/S1600536808010805/si2072sup1.cif

Structure factors: contains datablocks I. DOI: 10.1107/S1600536808010805/si2072Isup2.hkl

Additional supplementary materials:  crystallographic information; 3D view; checkCIF report

## Figures and Tables

**Table 1:** Selected bond lengths (Å)

Pb1—O1	2.582 (5)
Pb1—O2	2.824 (5)
Pb1—O5^i^	2.818 (6)
Pb1—N1	2.672 (6)
Pb1—N2	2.570 (6)
Pb1—N5	2.612 (6)
Pb1—N6	2.506 (6)

Symmetry code: (i) 
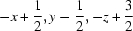
.

**Table 2:** Hydrogen-bond geometry (Å, °)

*D*—H⋯*A*	*D*—H	H⋯*A*	*D*⋯*A*	*D*—H⋯*A*
N3—H3N⋯O2^ii^	0.86	1.98	2.82 (1)	166
N7—H7N⋯O4^iii^	0.86	1.97	2.81 (1)	166
O1*W*—H1*W*1⋯N4	0.82	2.00	2.82 (1)	174
O1*W*—H1*W*2⋯O6^iv^	0.82	2.37	2.57 (1)	95
O2*W*—H2*W*1⋯N8	0.82	2.00	2.79 (1)	160
O2*W*—H2*W*2⋯O3^v^	0.82	2.27	3.06 (1)	160

Symmetry codes: (ii) 
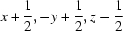
; (iii) 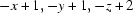; (iv) 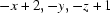; (v) 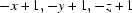.

**Fig. 1.**
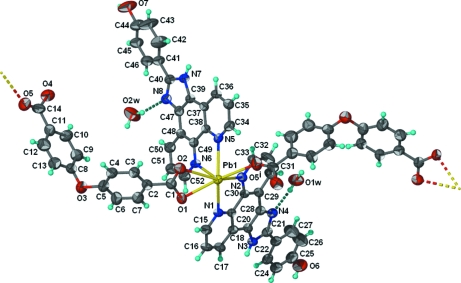
Thermal ellipsoid plot of a portion of the chain structure of Pb(C19H12N4O)2(C14H8O5).2H2O; displacement ellipsoids are drawn at the 50% probability level, and H atoms as spheres of arbitrary radius. Symmetry codes are given in Table 1.

**Fig. 2.**
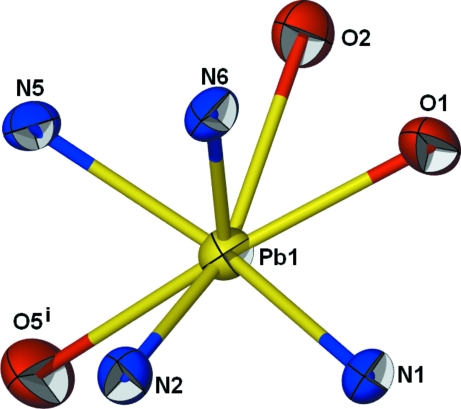
Seven-coordinate environment of lead.

## supplementary crystallographic information

### Comment

The 4-(1*H*-1,3,7,8-tetraazacyclopenta[*l*]phenanthren-2-yl)phenol *N*-heterocycle furnishes adducts with metal dicarboxylates (Xu *et al.*, 2008).

The carboxylate dianion in the title chain compound uses one carboxyl –CO_2_ group to *O*,*O*'-chelate to the lead(II) atom and uses its other carboxyl end to bind to another lead atom in a unidentate manner. The lead atom exists in an undefined seven-coordinate geometry; the Pb—O distances range between 2.582 (5) and 2.824 (5) Å, and the Pb—N distances vary between 2.506 (6) and 2.672 (6) Å (Table 1). The lone-pair is stereochemically active. Adjacent chains are linked by intermolecular O—H···N, N—H···O and O—H···O hydrogen bonds (Table 2) that involve the lattice water molecules to form a three-dimensional network.

### Experimental

Lead(II) nitrate (0.1 mmol), 1,4-oxobis(benzoic acid), 4-(1*H*-1,3,7,8-tetraazacyclopenta[*l*]phenanthren-2-yl)phenol (0.1 mmol) and water (14 ml) were heated in a 23 ml, Teflon-lined, stainless-steel Parr bomb at 458 K for 3 days. Crystals were obtained in 30% yield.

### Refinement

The carbon- and nitrogen-bound H atoms were placed in calculated positions [C—H 0.93, N—H 0.86, O—H 0.82 Å and *U*_iso_(H)= 1.2*U*_eq_(C, N, O)], and were included in the refinement in the riding-model approximation.

### Crystal data

**Table:**

[Pb(C_14_H_8_O_5_)(C_19_H_12_N_4_O)_2_]·2H_2_O	*F*_000_ = 2232
*M**_r_* = 1124.08	*D*_x_ = 1.709 Mg m^−^^3^
Monoclinic, *P*2_1_/*n*	Mo *K*α radiation λ = 0.71073 Å
Hall symbol: -P 2yn	Cell parameters from 24960 reflections
*a* = 10.767 (4) Å	θ = 3.0–27.5º
*b* = 29.916 (7) Å	µ = 3.93 mm^−^^1^
*c* = 13.688 (4) Å	*T* = 295 (2) K
β = 97.70 (1)º	Block, colorless
*V* = 4369 (2) Å^3^	0.33 × 0.24 × 0.21 mm
*Z* = 4	

### Data collection

**Table:**

Rigaku R-AXIS RAPID diffractometer	9977 independent reflections
Radiation source: fine-focus sealed tube	6235 reflections with *I* > 2σ(*I*)
Monochromator: graphite	*R*_int_ = 0.063
Detector resolution: 10 pixels mm^-1^	θ_max_ = 27.5º
*T* = 295(2) K	θ_min_ = 3.0º
ω scans	*h* = −13→13
Absorption correction: Multi-scan(ABSCOR; Higashi, 1995)	*k* = −38→38
*T*_min_ = 0.188, *T*_max_ = 0.492	*l* = −17→17
42355 measured reflections	

### Refinement

**Table:**

Refinement on *F*^2^	Secondary atom site location: difference Fourier map
Least-squares matrix: full	Hydrogen site location: inferred from neighbouring sites
*R*[*F*^2^ > 2σ(*F*^2^)] = 0.055	H-atom parameters constrained
*wR*(*F*^2^) = 0.168	*w* = 1/[σ^2^(*F*_o_^2^) + (0.0814*P*)^2^ + 9.1333*P*] where *P* = (*F*_o_^2^ + 2*F*_c_^2^)/3
*S* = 1.02	(Δ/σ)_max_ = 0.001
9977 reflections	Δρ_max_ = 1.52 e Å^−^^3^
631 parameters	Δρ_min_ = −1.09 e Å^−^^3^
Primary atom site location: structure-invariant direct methods	Extinction correction: none

### Fractional atomic coordinates and isotropic or equivalent isotropic displacement parameters (Å^2^)

**Table:**

	*x*	*y*	*z*	*U*_iso_*/*U*_eq_	
Pb1	0.22221 (3)	0.252248 (8)	0.632884 (19)	0.04982 (12)	
O1	0.1768 (7)	0.31678 (17)	0.5080 (4)	0.0838 (19)	
O2	0.1246 (6)	0.33913 (18)	0.6496 (4)	0.0704 (15)	
O3	0.2197 (8)	0.5257 (2)	0.4374 (5)	0.099 (2)	
O4	0.4930 (7)	0.6379 (2)	0.7966 (5)	0.094 (2)	
O5	0.3496 (9)	0.6846 (2)	0.7428 (5)	0.114 (3)	
O6	1.0871 (7)	0.0039 (2)	0.2469 (5)	0.092 (2)	
H6O	1.0994	0.0068	0.1894	0.138*	
O7	0.9813 (8)	0.5095 (3)	1.2543 (5)	0.124 (3)	
H7O	1.0442	0.5223	1.2408	0.186*	
O1W	0.8513 (7)	0.0694 (2)	0.6615 (5)	0.097 (2)	
H1W1	0.8047	0.0778	0.6127	0.145*	
H1W2	0.8102	0.0643	0.7066	0.145*	
O2W	0.8223 (7)	0.4435 (3)	0.7773 (5)	0.128 (3)	
H2W1	0.7757	0.4259	0.8005	0.191*	
H2W2	0.7958	0.4477	0.7191	0.191*	
N1	0.3196 (6)	0.2288 (2)	0.4707 (4)	0.0541 (15)	
N2	0.4077 (6)	0.19747 (19)	0.6534 (4)	0.0560 (15)	
N3	0.6337 (6)	0.13120 (19)	0.3461 (4)	0.0542 (15)	
H3N	0.6276	0.1356	0.2836	0.065*	
N4	0.7003 (6)	0.10439 (19)	0.4949 (4)	0.0554 (15)	
N5	0.3031 (6)	0.27173 (18)	0.8164 (4)	0.0491 (14)	
N6	0.4143 (6)	0.30027 (19)	0.6616 (4)	0.0512 (14)	
N7	0.6079 (6)	0.36530 (19)	1.0242 (4)	0.0516 (14)	
H7N	0.5899	0.3632	1.0834	0.062*	
N8	0.7061 (6)	0.38671 (19)	0.8986 (4)	0.0536 (15)	
C1	0.1535 (8)	0.3474 (3)	0.5655 (6)	0.065 (2)	
C2	0.1686 (8)	0.3954 (3)	0.5314 (6)	0.061 (2)	
C3	0.1180 (8)	0.4303 (2)	0.5783 (6)	0.066 (2)	
H3	0.0716	0.4248	0.6297	0.079*	
C4	0.1373 (9)	0.4749 (3)	0.5474 (6)	0.069 (2)	
H4	0.1044	0.4988	0.5788	0.082*	
C5	0.2045 (9)	0.4823 (3)	0.4712 (7)	0.072 (2)	
C6	0.2548 (10)	0.4470 (3)	0.4242 (7)	0.084 (3)	
H6	0.2991	0.4528	0.3717	0.101*	
C7	0.2405 (9)	0.4042 (3)	0.4532 (6)	0.075 (3)	
H7	0.2772	0.3808	0.4225	0.090*	
C8	0.2650 (11)	0.5560 (3)	0.5087 (6)	0.079 (3)	
C9	0.3789 (11)	0.5484 (3)	0.5651 (7)	0.098 (3)	
H9	0.4252	0.5230	0.5548	0.117*	
C10	0.4246 (11)	0.5793 (3)	0.6381 (7)	0.096 (3)	
H10	0.5000	0.5737	0.6779	0.115*	
C11	0.3572 (9)	0.6186 (3)	0.6515 (6)	0.070 (2)	
C12	0.2442 (9)	0.6260 (4)	0.5880 (7)	0.084 (3)	
H12	0.1993	0.6523	0.5934	0.101*	
C13	0.2011 (9)	0.5948 (3)	0.5191 (7)	0.077 (2)	
H13	0.1264	0.6001	0.4781	0.093*	
C14	0.4078 (11)	0.6516 (3)	0.7334 (7)	0.090 (3)	
C15	0.2765 (9)	0.2453 (2)	0.3820 (6)	0.065 (2)	
H15	0.2113	0.2659	0.3768	0.078*	
C16	0.3252 (10)	0.2329 (3)	0.2974 (5)	0.072 (2)	
H16	0.2946	0.2457	0.2371	0.086*	
C17	0.4159 (8)	0.2026 (3)	0.3029 (5)	0.061 (2)	
H17	0.4484	0.1937	0.2463	0.073*	
C18	0.4623 (7)	0.1841 (2)	0.3943 (5)	0.0534 (18)	
C19	0.4116 (7)	0.1985 (2)	0.4769 (5)	0.0478 (16)	
C20	0.5610 (7)	0.1511 (2)	0.4104 (5)	0.0522 (17)	
C21	0.7153 (8)	0.1039 (2)	0.3996 (6)	0.0575 (19)	
C22	0.8102 (8)	0.0774 (3)	0.3582 (6)	0.060 (2)	
C23	0.8326 (9)	0.0832 (2)	0.2636 (6)	0.066 (2)	
H23	0.7873	0.1044	0.2240	0.079*	
C24	0.9235 (9)	0.0574 (3)	0.2253 (6)	0.069 (2)	
H24	0.9353	0.0603	0.1596	0.083*	
C25	0.9933 (9)	0.0285 (3)	0.2840 (7)	0.076 (2)	
C26	0.9773 (12)	0.0230 (4)	0.3790 (7)	0.116 (5)	
H26	1.0265	0.0030	0.4194	0.139*	
C27	0.8852 (12)	0.0481 (4)	0.4143 (8)	0.110 (4)	
H27	0.8739	0.0447	0.4801	0.132*	
C28	0.6054 (7)	0.1341 (2)	0.5018 (5)	0.0519 (17)	
C29	0.5571 (8)	0.1494 (2)	0.5886 (5)	0.0532 (18)	
C30	0.4597 (7)	0.1814 (2)	0.5749 (5)	0.0475 (16)	
C31	0.5963 (9)	0.1339 (3)	0.6837 (6)	0.075 (3)	
H31	0.6577	0.1119	0.6944	0.090*	
C32	0.5450 (10)	0.1509 (3)	0.7599 (6)	0.088 (3)	
H32	0.5733	0.1418	0.8240	0.106*	
C33	0.4502 (9)	0.1819 (3)	0.7424 (6)	0.072 (2)	
H33	0.4139	0.1925	0.7958	0.087*	
C34	0.2457 (8)	0.2584 (2)	0.8911 (6)	0.0573 (19)	
H34	0.1771	0.2394	0.8778	0.069*	
C35	0.2824 (8)	0.2712 (3)	0.9875 (6)	0.062 (2)	
H35	0.2385	0.2610	1.0372	0.074*	
C36	0.3824 (7)	0.2986 (2)	1.0100 (5)	0.0542 (18)	
H36	0.4086	0.3072	1.0749	0.065*	
C37	0.4461 (6)	0.3140 (2)	0.9323 (5)	0.0439 (15)	
C38	0.4027 (7)	0.2994 (2)	0.8353 (5)	0.0445 (15)	
C39	0.5495 (7)	0.3434 (2)	0.9428 (5)	0.0487 (16)	
C40	0.6998 (7)	0.3911 (2)	0.9936 (5)	0.0534 (18)	
C41	0.7755 (7)	0.4217 (3)	1.0608 (6)	0.0593 (19)	
C42	0.7432 (11)	0.4316 (4)	1.1507 (8)	0.116 (5)	
H42	0.6739	0.4175	1.1707	0.140*	
C43	0.8099 (13)	0.4621 (5)	1.2140 (9)	0.133 (6)	
H43	0.7826	0.4698	1.2733	0.160*	
C44	0.9163 (9)	0.4804 (3)	1.1872 (7)	0.083 (3)	
C45	0.9535 (8)	0.4709 (3)	1.1008 (6)	0.073 (2)	
H45	1.0265	0.4834	1.0834	0.087*	
C46	0.8813 (8)	0.4416 (3)	1.0360 (6)	0.072 (2)	
H46	0.9059	0.4356	0.9748	0.086*	
C47	0.6115 (6)	0.3568 (2)	0.8667 (5)	0.0493 (16)	
C48	0.5710 (7)	0.3414 (2)	0.7678 (5)	0.0496 (16)	
C49	0.4643 (7)	0.3140 (2)	0.7525 (5)	0.0477 (16)	
C50	0.6268 (8)	0.3542 (3)	0.6863 (6)	0.066 (2)	
H50	0.6980	0.3722	0.6944	0.079*	
C51	0.5767 (9)	0.3404 (3)	0.5946 (6)	0.074 (2)	
H51	0.6132	0.3486	0.5394	0.089*	
C52	0.4692 (9)	0.3134 (3)	0.5850 (6)	0.068 (2)	
H52	0.4349	0.3043	0.5222	0.082*	

### Atomic displacement parameters (Å^2^)

**Table:**

	*U*^11^	*U*^22^	*U*^33^	*U*^12^	*U*^13^	*U*^23^
Pb1	0.06011 (19)	0.04550 (16)	0.04314 (16)	−0.00172 (12)	0.00430 (12)	−0.00412 (12)
O1	0.151 (6)	0.047 (3)	0.056 (3)	0.019 (3)	0.023 (4)	0.001 (3)
O2	0.094 (4)	0.069 (3)	0.053 (3)	0.002 (3)	0.026 (3)	0.002 (3)
O3	0.154 (7)	0.065 (4)	0.074 (4)	−0.025 (4)	0.000 (4)	0.002 (3)
O4	0.112 (6)	0.090 (5)	0.074 (4)	−0.002 (4)	−0.007 (4)	−0.011 (4)
O5	0.199 (9)	0.065 (4)	0.080 (5)	0.022 (5)	0.032 (5)	−0.011 (4)
O6	0.108 (5)	0.098 (5)	0.077 (4)	0.016 (4)	0.039 (4)	−0.020 (4)
O7	0.129 (7)	0.160 (7)	0.088 (5)	−0.084 (6)	0.031 (5)	−0.058 (5)
O1W	0.109 (5)	0.111 (5)	0.067 (4)	0.035 (4)	0.003 (4)	−0.012 (4)
O2W	0.120 (6)	0.166 (8)	0.087 (5)	−0.080 (6)	−0.021 (4)	0.042 (5)
N1	0.075 (4)	0.050 (3)	0.036 (3)	−0.001 (3)	0.003 (3)	−0.004 (3)
N2	0.077 (4)	0.051 (3)	0.042 (3)	0.009 (3)	0.013 (3)	−0.001 (3)
N3	0.079 (4)	0.052 (3)	0.034 (3)	0.000 (3)	0.015 (3)	−0.004 (3)
N4	0.071 (4)	0.050 (3)	0.046 (3)	0.007 (3)	0.013 (3)	−0.007 (3)
N5	0.062 (4)	0.042 (3)	0.044 (3)	−0.008 (3)	0.012 (3)	−0.007 (3)
N6	0.065 (4)	0.052 (3)	0.036 (3)	−0.005 (3)	0.005 (3)	−0.007 (3)
N7	0.061 (4)	0.051 (3)	0.042 (3)	−0.005 (3)	0.004 (3)	−0.006 (3)
N8	0.056 (4)	0.052 (3)	0.053 (4)	−0.005 (3)	0.006 (3)	−0.002 (3)
C1	0.086 (6)	0.054 (4)	0.056 (5)	0.011 (4)	0.012 (4)	0.000 (4)
C2	0.067 (5)	0.058 (4)	0.054 (5)	−0.002 (4)	0.000 (4)	0.004 (4)
C3	0.084 (6)	0.053 (4)	0.060 (5)	0.008 (4)	0.004 (4)	−0.004 (4)
C4	0.088 (6)	0.057 (4)	0.060 (5)	0.005 (4)	0.005 (5)	−0.006 (4)
C5	0.088 (6)	0.055 (4)	0.072 (6)	−0.009 (4)	0.011 (5)	−0.001 (4)
C6	0.115 (8)	0.064 (5)	0.078 (6)	−0.008 (5)	0.027 (6)	0.006 (5)
C7	0.099 (7)	0.067 (5)	0.062 (5)	−0.014 (5)	0.027 (5)	−0.001 (4)
C8	0.124 (9)	0.062 (5)	0.049 (5)	−0.018 (5)	0.006 (5)	0.003 (4)
C9	0.135 (10)	0.073 (6)	0.076 (6)	0.026 (6)	−0.018 (6)	0.001 (5)
C10	0.128 (9)	0.074 (6)	0.077 (6)	0.021 (6)	−0.015 (6)	−0.018 (5)
C11	0.090 (7)	0.060 (5)	0.063 (5)	0.001 (4)	0.022 (5)	0.004 (4)
C12	0.081 (7)	0.091 (7)	0.079 (6)	0.008 (5)	0.007 (5)	0.002 (6)
C13	0.076 (6)	0.080 (6)	0.074 (6)	−0.007 (5)	0.003 (5)	−0.002 (5)
C14	0.133 (9)	0.078 (6)	0.050 (5)	0.044 (6)	−0.021 (5)	−0.011 (5)
C15	0.083 (6)	0.062 (5)	0.047 (4)	0.009 (4)	−0.001 (4)	0.000 (4)
C16	0.109 (7)	0.069 (5)	0.032 (4)	0.008 (5)	−0.004 (4)	−0.003 (4)
C17	0.080 (6)	0.066 (5)	0.036 (4)	0.000 (4)	0.009 (4)	0.003 (3)
C18	0.073 (5)	0.051 (4)	0.039 (4)	−0.010 (3)	0.015 (4)	−0.006 (3)
C19	0.064 (5)	0.041 (3)	0.036 (3)	−0.004 (3)	0.001 (3)	−0.001 (3)
C20	0.073 (5)	0.046 (3)	0.039 (4)	−0.002 (3)	0.011 (3)	0.001 (3)
C21	0.076 (5)	0.051 (4)	0.048 (4)	0.004 (4)	0.014 (4)	−0.011 (3)
C22	0.077 (6)	0.056 (4)	0.049 (4)	0.000 (4)	0.017 (4)	−0.010 (4)
C23	0.091 (6)	0.051 (4)	0.058 (5)	−0.002 (4)	0.018 (4)	−0.012 (4)
C24	0.087 (6)	0.069 (5)	0.056 (5)	0.002 (5)	0.029 (5)	−0.012 (4)
C25	0.094 (7)	0.065 (5)	0.074 (6)	0.010 (5)	0.030 (5)	−0.015 (5)
C26	0.158 (12)	0.124 (9)	0.073 (7)	0.084 (8)	0.043 (7)	0.016 (6)
C27	0.160 (12)	0.112 (8)	0.066 (6)	0.057 (8)	0.042 (7)	0.001 (6)
C28	0.067 (5)	0.049 (4)	0.041 (4)	0.001 (3)	0.014 (3)	−0.005 (3)
C29	0.075 (5)	0.048 (4)	0.038 (4)	0.006 (3)	0.010 (3)	0.001 (3)
C30	0.065 (5)	0.045 (3)	0.033 (3)	−0.005 (3)	0.008 (3)	−0.003 (3)
C31	0.098 (7)	0.081 (6)	0.048 (5)	0.035 (5)	0.020 (4)	0.010 (4)
C32	0.117 (8)	0.102 (7)	0.045 (5)	0.047 (6)	0.013 (5)	0.018 (5)
C33	0.099 (7)	0.076 (5)	0.043 (4)	0.033 (5)	0.013 (4)	0.004 (4)
C34	0.062 (5)	0.053 (4)	0.058 (5)	−0.012 (3)	0.011 (4)	−0.011 (4)
C35	0.083 (6)	0.059 (4)	0.046 (4)	−0.005 (4)	0.020 (4)	0.002 (4)
C36	0.068 (5)	0.058 (4)	0.036 (4)	−0.002 (4)	0.004 (3)	−0.003 (3)
C37	0.049 (4)	0.044 (3)	0.038 (3)	0.004 (3)	0.003 (3)	0.003 (3)
C38	0.057 (4)	0.038 (3)	0.038 (3)	0.002 (3)	0.006 (3)	−0.004 (3)
C39	0.060 (4)	0.046 (3)	0.040 (4)	−0.003 (3)	0.005 (3)	−0.004 (3)
C40	0.059 (5)	0.051 (4)	0.048 (4)	−0.005 (3)	0.000 (3)	−0.004 (3)
C41	0.060 (5)	0.069 (5)	0.048 (4)	−0.011 (4)	0.002 (4)	−0.012 (4)
C42	0.128 (10)	0.151 (11)	0.077 (7)	−0.090 (8)	0.038 (7)	−0.027 (7)
C43	0.151 (12)	0.174 (12)	0.084 (8)	−0.102 (10)	0.049 (8)	−0.067 (8)
C44	0.076 (6)	0.101 (7)	0.070 (6)	−0.040 (5)	0.008 (5)	−0.024 (5)
C45	0.072 (6)	0.090 (6)	0.056 (5)	−0.027 (5)	0.005 (4)	−0.012 (5)
C46	0.083 (6)	0.079 (5)	0.052 (5)	−0.019 (5)	0.007 (4)	−0.007 (4)
C47	0.048 (4)	0.046 (3)	0.053 (4)	0.004 (3)	0.002 (3)	0.001 (3)
C48	0.061 (5)	0.046 (4)	0.043 (4)	0.000 (3)	0.011 (3)	0.001 (3)
C49	0.054 (4)	0.043 (3)	0.046 (4)	0.002 (3)	0.009 (3)	0.001 (3)
C50	0.073 (6)	0.064 (5)	0.059 (5)	−0.018 (4)	0.005 (4)	−0.002 (4)
C51	0.096 (7)	0.078 (6)	0.051 (5)	−0.023 (5)	0.021 (5)	0.000 (4)
C52	0.090 (6)	0.070 (5)	0.046 (4)	−0.017 (5)	0.014 (4)	−0.003 (4)

### Geometric parameters (Å, °)

**Table:**

Pb1—O1	2.582 (5)	C13—H13	0.9300
Pb1—O2	2.824 (5)	C15—C16	1.384 (12)
Pb1—O5^i^	2.818 (6)	C15—H15	0.9300
Pb1—N1	2.672 (6)	C16—C17	1.327 (11)
Pb1—N2	2.570 (6)	C16—H16	0.9300
Pb1—N5	2.612 (6)	C17—C18	1.398 (10)
Pb1—N6	2.506 (6)	C17—H17	0.9300
O1—C1	1.255 (9)	C18—C19	1.388 (9)
O2—C1	1.257 (9)	C18—C20	1.446 (10)
O3—C8	1.371 (10)	C19—C30	1.464 (9)
O3—C5	1.397 (9)	C20—C28	1.376 (9)
O4—C14	1.243 (10)	C21—C22	1.466 (11)
O5—C14	1.184 (10)	C22—C27	1.357 (12)
O6—C25	1.398 (10)	C22—C23	1.360 (10)
O6—H6O	0.8200	C23—C24	1.402 (11)
O7—C44	1.385 (10)	C23—H23	0.9300
O7—H7O	0.8201	C24—C25	1.339 (12)
O1W—H1W1	0.8199	C24—H24	0.9300
O1W—H1W2	0.8200	C25—C26	1.344 (12)
O2W—H2W1	0.8199	C26—C27	1.383 (13)
O2W—H2W2	0.8201	C26—H26	0.9300
N1—C15	1.335 (10)	C27—H27	0.9300
N1—C19	1.339 (9)	C28—C29	1.434 (9)
N2—C33	1.327 (9)	C29—C31	1.393 (10)
N2—C30	1.364 (9)	C29—C30	1.413 (10)
N3—C21	1.343 (10)	C31—C32	1.344 (11)
N3—C20	1.387 (9)	C31—H31	0.9300
N3—H3N	0.8600	C32—C33	1.377 (11)
N4—C21	1.335 (9)	C32—H32	0.9300
N4—C28	1.367 (9)	C33—H33	0.9300
N5—C34	1.325 (10)	C34—C35	1.381 (11)
N5—C38	1.353 (8)	C34—H34	0.9300
N6—C52	1.330 (9)	C35—C36	1.356 (10)
N6—C49	1.352 (8)	C35—H35	0.9300
N7—C40	1.364 (9)	C36—C37	1.417 (9)
N7—C39	1.371 (8)	C36—H36	0.9300
N7—H7N	0.8600	C37—C39	1.410 (9)
N8—C40	1.318 (9)	C37—C38	1.417 (9)
N8—C47	1.382 (9)	C38—C49	1.454 (9)
C1—C2	1.524 (11)	C39—C47	1.371 (10)
C2—C3	1.376 (11)	C40—C41	1.466 (10)
C2—C7	1.427 (11)	C41—C42	1.355 (12)
C3—C4	1.423 (11)	C41—C46	1.368 (11)
C3—H3	0.9300	C42—C43	1.390 (13)
C4—C5	1.364 (12)	C42—H42	0.9300
C4—H4	0.9300	C43—C44	1.363 (13)
C5—C6	1.383 (12)	C43—H43	0.9300
C6—C7	1.357 (11)	C44—C45	1.330 (12)
C6—H6	0.9300	C45—C46	1.405 (11)
C7—H7	0.9300	C45—H45	0.9300
C8—C13	1.369 (13)	C46—H46	0.9300
C8—C9	1.378 (13)	C47—C48	1.440 (9)
C9—C10	1.399 (13)	C48—C50	1.390 (11)
C9—H9	0.9300	C48—C49	1.404 (10)
C10—C11	1.407 (12)	C50—C51	1.363 (11)
C10—H10	0.9300	C50—H50	0.9300
C11—C12	1.415 (13)	C51—C52	1.403 (12)
C11—C14	1.539 (12)	C51—H51	0.9300
C12—C13	1.364 (13)	C52—H52	0.9300
C12—H12	0.9300		
			
N6—Pb1—N2	74.7 (2)	N1—C19—C18	121.8 (6)
N6—Pb1—O1	75.8 (2)	N1—C19—C30	117.6 (6)
N2—Pb1—O1	128.6 (2)	C18—C19—C30	120.6 (7)
N6—Pb1—N5	63.86 (18)	C28—C20—N3	105.2 (6)
N2—Pb1—N5	83.28 (19)	C28—C20—C18	123.3 (6)
O1—Pb1—N5	118.63 (18)	N3—C20—C18	131.4 (6)
N6—Pb1—N1	82.70 (18)	N4—C21—N3	111.7 (6)
N2—Pb1—N1	62.47 (18)	N4—C21—C22	124.4 (7)
O1—Pb1—N1	72.93 (18)	N3—C21—C22	123.9 (7)
N5—Pb1—N1	137.72 (19)	C27—C22—C23	117.0 (8)
N6—Pb1—O5^i^	127.2 (2)	C27—C22—C21	121.6 (8)
N2—Pb1—O5^i^	75.4 (2)	C23—C22—C21	121.4 (8)
O1—Pb1—O5^i^	153.4 (3)	C22—C23—C24	120.4 (8)
N5—Pb1—O5^i^	70.28 (19)	C22—C23—H23	119.8
N1—Pb1—O5^i^	118.9 (2)	C24—C23—H23	119.8
N6—Pb1—O2	76.57 (18)	C25—C24—C23	119.7 (8)
N2—Pb1—O2	150.48 (19)	C25—C24—H24	120.1
O1—Pb1—O2	47.62 (16)	C23—C24—H24	120.1
N5—Pb1—O2	78.26 (17)	C24—C25—C26	121.6 (9)
N1—Pb1—O2	120.01 (17)	C24—C25—O6	119.8 (8)
O5^i^—Pb1—O2	118.49 (19)	C26—C25—O6	118.6 (9)
C1—O1—Pb1	99.6 (5)	C25—C26—C27	117.5 (10)
C1—O2—Pb1	88.1 (4)	C25—C26—H26	121.2
C8—O3—C5	115.1 (7)	C27—C26—H26	121.2
C25—O6—H6O	120.7	C22—C27—C26	123.6 (9)
C44—O7—H7O	120.1	C22—C27—H27	118.2
H1W1—O1W—H1W2	109.8	C26—C27—H27	118.2
H2W1—O2W—H2W2	108.8	N4—C28—C20	110.4 (6)
C15—N1—C19	118.1 (6)	N4—C28—C29	128.5 (7)
C15—N1—Pb1	122.1 (5)	C20—C28—C29	121.0 (7)
C19—N1—Pb1	119.7 (4)	C31—C29—C30	118.4 (7)
C33—N2—C30	118.1 (6)	C31—C29—C28	124.7 (7)
C33—N2—Pb1	119.4 (5)	C30—C29—C28	116.8 (6)
C30—N2—Pb1	122.3 (5)	N2—C30—C29	120.7 (6)
C21—N3—C20	107.3 (6)	N2—C30—C19	117.8 (6)
C21—N3—H3N	126.3	C29—C30—C19	121.5 (6)
C20—N3—H3N	126.3	C32—C31—C29	119.7 (8)
C21—N4—C28	105.4 (6)	C32—C31—H31	120.1
C34—N5—C38	118.5 (6)	C29—C31—H31	120.1
C34—N5—Pb1	123.0 (5)	C31—C32—C33	119.4 (8)
C38—N5—Pb1	118.3 (4)	C31—C32—H32	120.3
C52—N6—C49	118.1 (7)	C33—C32—H32	120.3
C52—N6—Pb1	119.4 (5)	N2—C33—C32	123.6 (8)
C49—N6—Pb1	122.5 (5)	N2—C33—H33	118.2
C40—N7—C39	107.1 (6)	C32—C33—H33	118.2
C40—N7—H7N	126.5	N5—C34—C35	123.4 (7)
C39—N7—H7N	126.5	N5—C34—H34	118.3
C40—N8—C47	104.0 (6)	C35—C34—H34	118.3
O2—C1—O1	121.6 (7)	C36—C35—C34	120.2 (7)
O2—C1—C2	121.0 (7)	C36—C35—H35	119.9
O1—C1—C2	117.3 (7)	C34—C35—H35	119.9
C3—C2—C7	119.9 (8)	C35—C36—C37	118.4 (6)
C3—C2—C1	120.5 (8)	C35—C36—H36	120.8
C7—C2—C1	119.5 (8)	C37—C36—H36	120.8
C2—C3—C4	119.3 (8)	C39—C37—C38	116.3 (6)
C2—C3—H3	120.3	C39—C37—C36	125.5 (6)
C4—C3—H3	120.3	C38—C37—C36	118.2 (6)
C5—C4—C3	119.5 (8)	N5—C38—C37	121.4 (6)
C5—C4—H4	120.2	N5—C38—C49	117.9 (6)
C3—C4—H4	120.2	C37—C38—C49	120.8 (6)
C4—C5—C6	120.9 (8)	N7—C39—C47	105.1 (6)
C4—C5—O3	120.1 (8)	N7—C39—C37	130.6 (6)
C6—C5—O3	119.0 (9)	C47—C39—C37	124.3 (6)
C7—C6—C5	121.1 (9)	N8—C40—N7	112.5 (6)
C7—C6—H6	119.4	N8—C40—C41	125.7 (7)
C5—C6—H6	119.4	N7—C40—C41	121.7 (7)
C6—C7—C2	119.2 (9)	C42—C41—C46	117.0 (8)
C6—C7—H7	120.4	C42—C41—C40	121.2 (8)
C2—C7—H7	120.4	C46—C41—C40	121.8 (7)
O3—C8—C13	119.9 (10)	C41—C42—C43	122.4 (9)
O3—C8—C9	119.8 (9)	C41—C42—H42	118.8
C13—C8—C9	120.2 (9)	C43—C42—H42	118.8
C8—C9—C10	119.6 (10)	C44—C43—C42	118.6 (10)
C8—C9—H9	120.2	C44—C43—H43	120.7
C10—C9—H9	120.2	C42—C43—H43	120.7
C9—C10—C11	120.4 (10)	C45—C44—C43	121.1 (9)
C9—C10—H10	119.8	C45—C44—O7	122.6 (8)
C11—C10—H10	119.8	C43—C44—O7	116.3 (9)
C10—C11—C12	117.9 (9)	C44—C45—C46	119.2 (8)
C10—C11—C14	119.6 (9)	C44—C45—H45	120.4
C12—C11—C14	122.5 (8)	C46—C45—H45	120.4
C13—C12—C11	120.2 (9)	C41—C46—C45	121.6 (8)
C13—C12—H12	119.9	C41—C46—H46	119.2
C11—C12—H12	119.9	C45—C46—H46	119.2
C8—C13—C12	121.6 (9)	C39—C47—N8	111.3 (6)
C8—C13—H13	119.2	C39—C47—C48	120.2 (6)
C12—C13—H13	119.2	N8—C47—C48	128.4 (7)
O5—C14—O4	123.5 (9)	C50—C48—C49	118.3 (7)
O5—C14—C11	118.2 (9)	C50—C48—C47	123.8 (7)
O4—C14—C11	116.7 (8)	C49—C48—C47	117.8 (6)
N1—C15—C16	122.5 (8)	N6—C49—C48	122.1 (6)
N1—C15—H15	118.7	N6—C49—C38	117.4 (6)
C16—C15—H15	118.7	C48—C49—C38	120.5 (6)
C17—C16—C15	119.6 (8)	C51—C50—C48	119.7 (8)
C17—C16—H16	120.2	C51—C50—H50	120.2
C15—C16—H16	120.2	C48—C50—H50	120.2
C16—C17—C18	119.5 (7)	C50—C51—C52	118.8 (8)
C16—C17—H17	120.2	C50—C51—H51	120.6
C18—C17—H17	120.2	C52—C51—H51	120.6
C19—C18—C17	118.3 (7)	N6—C52—C51	123.0 (7)
C19—C18—C20	116.7 (6)	N6—C52—H52	118.5
C17—C18—C20	125.0 (7)	C51—C52—H52	118.5
			
N6—Pb1—O1—C1	−75.1 (6)	C19—C18—C20—N3	−177.2 (7)
N2—Pb1—O1—C1	−131.5 (6)	C17—C18—C20—N3	2.1 (13)
N5—Pb1—O1—C1	−26.1 (6)	C28—N4—C21—N3	−0.9 (9)
N1—Pb1—O1—C1	−161.7 (6)	C28—N4—C21—C22	178.3 (7)
O5^i^—Pb1—O1—C1	77.3 (7)	C20—N3—C21—N4	0.3 (9)
O2—Pb1—O1—C1	9.6 (5)	C20—N3—C21—C22	−178.8 (7)
N6—Pb1—O2—C1	73.4 (5)	N4—C21—C22—C27	5.3 (14)
N2—Pb1—O2—C1	86.6 (6)	N3—C21—C22—C27	−175.7 (9)
O1—Pb1—O2—C1	−9.4 (5)	N4—C21—C22—C23	−170.4 (8)
N5—Pb1—O2—C1	139.0 (5)	N3—C21—C22—C23	8.6 (13)
N1—Pb1—O2—C1	0.2 (6)	C27—C22—C23—C24	4.4 (13)
O5^i^—Pb1—O2—C1	−161.3 (5)	C21—C22—C23—C24	−179.8 (8)
N6—Pb1—N1—C15	−102.2 (6)	C22—C23—C24—C25	−3.5 (13)
N2—Pb1—N1—C15	−178.7 (6)	C23—C24—C25—C26	1.0 (16)
O1—Pb1—N1—C15	−24.9 (6)	C23—C24—C25—O6	−177.7 (8)
N5—Pb1—N1—C15	−139.0 (6)	C24—C25—C26—C27	0.5 (19)
O5^i^—Pb1—N1—C15	129.1 (6)	O6—C25—C26—C27	179.1 (11)
O2—Pb1—N1—C15	−32.3 (6)	C23—C22—C27—C26	−3.0 (18)
N6—Pb1—N1—C19	80.2 (5)	C21—C22—C27—C26	−178.8 (11)
N2—Pb1—N1—C19	3.8 (5)	C25—C26—C27—C22	1(2)
O1—Pb1—N1—C19	157.5 (6)	C21—N4—C28—C20	1.1 (9)
N5—Pb1—N1—C19	43.4 (6)	C21—N4—C28—C29	−175.5 (8)
O5^i^—Pb1—N1—C19	−48.5 (6)	N3—C20—C28—N4	−0.9 (8)
O2—Pb1—N1—C19	150.1 (5)	C18—C20—C28—N4	−178.6 (6)
N6—Pb1—N2—C33	90.8 (7)	N3—C20—C28—C29	176.0 (7)
O1—Pb1—N2—C33	147.7 (6)	C18—C20—C28—C29	−1.7 (11)
N5—Pb1—N2—C33	26.1 (6)	N4—C28—C29—C31	−4.0 (13)
N1—Pb1—N2—C33	−179.5 (7)	C20—C28—C29—C31	179.7 (8)
O5^i^—Pb1—N2—C33	−45.2 (6)	N4—C28—C29—C30	178.4 (7)
O2—Pb1—N2—C33	77.5 (7)	C20—C28—C29—C30	2.2 (11)
N6—Pb1—N2—C30	−93.5 (6)	C33—N2—C30—C29	−0.8 (11)
O1—Pb1—N2—C30	−36.6 (6)	Pb1—N2—C30—C29	−176.5 (5)
N5—Pb1—N2—C30	−158.2 (6)	C33—N2—C30—C19	179.5 (7)
N1—Pb1—N2—C30	−3.8 (5)	Pb1—N2—C30—C19	3.8 (9)
O5^i^—Pb1—N2—C30	130.5 (6)	C31—C29—C30—N2	1.7 (11)
O2—Pb1—N2—C30	−106.8 (6)	C28—C29—C30—N2	179.4 (7)
N6—Pb1—N5—C34	178.3 (6)	C31—C29—C30—C19	−178.6 (7)
N2—Pb1—N5—C34	−105.6 (6)	C28—C29—C30—C19	−0.9 (10)
O1—Pb1—N5—C34	123.7 (6)	N1—C19—C30—N2	0.0 (10)
N1—Pb1—N5—C34	−140.3 (5)	C18—C19—C30—N2	178.7 (7)
O5^i^—Pb1—N5—C34	−28.7 (6)	N1—C19—C30—C29	−179.7 (7)
O2—Pb1—N5—C34	97.6 (6)	C18—C19—C30—C29	−1.0 (10)
N6—Pb1—N5—C38	3.3 (4)	C30—C29—C31—C32	−2.8 (14)
N2—Pb1—N5—C38	79.5 (5)	C28—C29—C31—C32	179.6 (9)
O1—Pb1—N5—C38	−51.2 (5)	C29—C31—C32—C33	3.0 (16)
N1—Pb1—N5—C38	44.7 (6)	C30—N2—C33—C32	1.0 (14)
O5^i^—Pb1—N5—C38	156.4 (5)	Pb1—N2—C33—C32	176.8 (8)
O2—Pb1—N5—C38	−77.3 (5)	C31—C32—C33—N2	−2.1 (17)
N2—Pb1—N6—C52	88.8 (6)	C38—N5—C34—C35	−0.2 (11)
O1—Pb1—N6—C52	−48.7 (6)	Pb1—N5—C34—C35	−175.1 (6)
N5—Pb1—N6—C52	178.8 (7)	N5—C34—C35—C36	−0.4 (13)
N1—Pb1—N6—C52	25.4 (6)	C34—C35—C36—C37	0.8 (11)
O5^i^—Pb1—N6—C52	146.4 (6)	C35—C36—C37—C39	178.4 (7)
O2—Pb1—N6—C52	−97.9 (6)	C35—C36—C37—C38	−0.7 (10)
N2—Pb1—N6—C49	−91.9 (5)	C34—N5—C38—C37	0.3 (10)
O1—Pb1—N6—C49	130.5 (6)	Pb1—N5—C38—C37	175.4 (5)
N5—Pb1—N6—C49	−1.9 (5)	C34—N5—C38—C49	−179.7 (6)
N1—Pb1—N6—C49	−155.3 (5)	Pb1—N5—C38—C49	−4.5 (8)
O5^i^—Pb1—N6—C49	−34.3 (6)	C39—C37—C38—N5	−179.1 (6)
O2—Pb1—N6—C49	81.4 (5)	C36—C37—C38—N5	0.2 (10)
Pb1—O2—C1—O1	17.0 (9)	C39—C37—C38—C49	0.9 (9)
Pb1—O2—C1—C2	−159.6 (8)	C36—C37—C38—C49	−179.9 (6)
Pb1—O1—C1—O2	−18.9 (10)	C40—N7—C39—C47	0.9 (8)
Pb1—O1—C1—C2	157.8 (6)	C40—N7—C39—C37	−177.5 (7)
O2—C1—C2—C3	−18.4 (13)	C38—C37—C39—N7	175.5 (7)
O1—C1—C2—C3	164.8 (8)	C36—C37—C39—N7	−3.7 (12)
O2—C1—C2—C7	158.8 (8)	C38—C37—C39—C47	−2.6 (10)
O1—C1—C2—C7	−18.0 (12)	C36—C37—C39—C47	178.2 (7)
C7—C2—C3—C4	0.5 (13)	C47—N8—C40—N7	0.8 (8)
C1—C2—C3—C4	177.7 (7)	C47—N8—C40—C41	−175.6 (7)
C2—C3—C4—C5	0.7 (13)	C39—N7—C40—N8	−1.1 (9)
C3—C4—C5—C6	−0.4 (14)	C39—N7—C40—C41	175.5 (7)
C3—C4—C5—O3	177.3 (8)	N8—C40—C41—C42	164.3 (10)
C8—O3—C5—C4	52.7 (13)	N7—C40—C41—C42	−11.8 (13)
C8—O3—C5—C6	−129.6 (10)	N8—C40—C41—C46	−15.6 (13)
C4—C5—C6—C7	−1.1 (16)	N7—C40—C41—C46	168.3 (8)
O3—C5—C6—C7	−178.8 (9)	C46—C41—C42—C43	3.0 (19)
C5—C6—C7—C2	2.3 (15)	C40—C41—C42—C43	−177.0 (12)
C3—C2—C7—C6	−2.0 (14)	C41—C42—C43—C44	−4(2)
C1—C2—C7—C6	−179.2 (9)	C42—C43—C44—C45	3(2)
C5—O3—C8—C13	−126.1 (10)	C42—C43—C44—O7	−178.0 (12)
C5—O3—C8—C9	58.3 (13)	C43—C44—C45—C46	0.4 (18)
O3—C8—C9—C10	−179.7 (10)	O7—C44—C45—C46	−178.9 (10)
C13—C8—C9—C10	4.6 (16)	C42—C41—C46—C45	0.2 (15)
C8—C9—C10—C11	−2.4 (17)	C40—C41—C46—C45	−179.9 (8)
C9—C10—C11—C12	−1.1 (16)	C44—C45—C46—C41	−1.9 (15)
C9—C10—C11—C14	178.9 (10)	N7—C39—C47—N8	−0.4 (8)
C10—C11—C12—C13	2.5 (14)	C37—C39—C47—N8	178.1 (6)
C14—C11—C12—C13	−177.5 (10)	N7—C39—C47—C48	−177.4 (6)
O3—C8—C13—C12	−178.9 (9)	C37—C39—C47—C48	1.0 (11)
C9—C8—C13—C12	−3.3 (16)	C40—N8—C47—C39	−0.2 (8)
C11—C12—C13—C8	−0.4 (15)	C40—N8—C47—C48	176.5 (7)
C10—C11—C14—O5	179.6 (11)	C39—C47—C48—C50	178.5 (7)
C12—C11—C14—O5	−0.4 (16)	N8—C47—C48—C50	2.0 (12)
C10—C11—C14—O4	−13.9 (15)	C39—C47—C48—C49	2.3 (10)
C12—C11—C14—O4	166.1 (10)	N8—C47—C48—C49	−174.1 (7)
C19—N1—C15—C16	−1.5 (12)	C52—N6—C49—C48	0.2 (11)
Pb1—N1—C15—C16	−179.1 (7)	Pb1—N6—C49—C48	−179.1 (5)
N1—C15—C16—C17	1.9 (14)	C52—N6—C49—C38	179.8 (7)
C15—C16—C17—C18	−1.0 (13)	Pb1—N6—C49—C38	0.5 (8)
C16—C17—C18—C19	−0.4 (12)	C50—C48—C49—N6	−0.7 (11)
C16—C17—C18—C20	−179.6 (8)	C47—C48—C49—N6	175.6 (6)
C15—N1—C19—C18	0.1 (11)	C50—C48—C49—C38	179.7 (7)
Pb1—N1—C19—C18	177.8 (5)	C47—C48—C49—C38	−4.0 (10)
C15—N1—C19—C30	178.8 (7)	N5—C38—C49—N6	2.7 (9)
Pb1—N1—C19—C30	−3.5 (8)	C37—C38—C49—N6	−177.2 (6)
C17—C18—C19—N1	0.8 (11)	N5—C38—C49—C48	−177.6 (6)
C20—C18—C19—N1	−179.8 (6)	C37—C38—C49—C48	2.4 (10)
C17—C18—C19—C30	−177.9 (7)	C49—C48—C50—C51	0.5 (12)
C20—C18—C19—C30	1.5 (10)	C47—C48—C50—C51	−175.6 (8)
C21—N3—C20—C28	0.3 (8)	C48—C50—C51—C52	0.1 (14)
C21—N3—C20—C18	177.8 (8)	C49—N6—C52—C51	0.5 (13)
C19—C18—C20—C28	−0.2 (11)	Pb1—N6—C52—C51	179.8 (7)
C17—C18—C20—C28	179.1 (7)	C50—C51—C52—N6	−0.7 (14)

Symmetry codes: (i) −*x*+1/2, *y*−1/2, −*z*+3/2.

### Hydrogen-bond geometry (Å, °)

**Table:**

*D*—H···*A*	*D*—H	H···*A*	*D*···*A*	*D*—H···*A*
N3—H3*N*···O2^ii^	0.86	1.98	2.82 (1)	166
N7—H7*N*···O4^iii^	0.86	1.97	2.81 (1)	166
O1*W*—H1*W*1···N4	0.82	2.00	2.82 (1)	174
O1*W*—H1*W*2···O6^iv^	0.82	2.37	2.57 (1)	95
O2*W*—H2*W*1···N8	0.82	2.00	2.79 (1)	160
O2*W*—H2*W*2···O3^v^	0.82	2.27	3.06 (1)	160

Symmetry codes: (ii) *x*+1/2, −*y*+1/2, *z*−1/2; (iii) −*x*+1, −*y*+1, −*z*+2; (iv) −*x*+2, −*y*, −*z*+1; (v) −*x*+1, −*y*+1, −*z*+1.
